# Knowledge and Attitudes of Youth Ice Hockey Players and Their Parents Toward Concussion in the United Kingdom

**DOI:** 10.7759/cureus.101130

**Published:** 2026-01-08

**Authors:** Christopher Fox, Aine K Jones

**Affiliations:** 1 Emergency Medicine, University Hospital of Wales, Cardiff, GBR; 2 Anesthesia, Swansea Bay University Health Board, Swansea, GBR

**Keywords:** adolescent ice hockey, concussion education, concussion prevention, ice hockey injury, sports related concussion

## Abstract

Introduction and aim

There is a lack of research investigating the knowledge and attitudes of youth ice hockey players and their parents toward concussion in the United Kingdom. This is a topic of increasing concern given the well-known risks associated with contact sports. Previous research in this area has primarily come from North America. The primary objective of our research was to evaluate the knowledge and attitudes regarding concussion among youth ice hockey players and their parents in the United Kingdom. The secondary objective was to examine whether increased knowledge was associated with improved attitudes toward concussion.

Methods

Using the Rosenbaum Concussion Knowledge and Attitudes Survey - Student Version (RoCKAS-ST), the research assessed both concussion knowledge (Concussion Knowledge Index {CKI}) and attitudes toward concussion management (Concussion Attitudes Index {CAI}) among youth players and their parents. The survey was distributed online to club secretaries to disseminate to their members. In addition, researchers attended local ice hockey matches and directly invited parents and players to participate in the survey. The collection period lasted for six weeks between October 12, 2025, and November 23, 2025. A total of 29 youth players and 55 parents met the inclusion criteria.

Results

Results revealed that both groups demonstrated relatively good levels of concussion knowledge, with parents scoring significantly higher than players (mean CKI: 19.69 vs. 17.86, p=0.0011). Similarly, parents exhibited safer attitudes toward concussion management (mean CAI: 62.62 vs. 57.41, p=0.0015). Notably, a strong positive correlation between knowledge and attitudes was observed among players (Pearson correlation coefficient 0.6213; 95% confidence interval 0.3299 to 0.8046), but not among parents (Pearson correlation coefficient 0.1852; 95% confidence interval -0.0842 to 0.4294). Notably, many players endorsed unsafe return-to-play decisions.

Conclusion

Despite overall positive findings, substantial variability and important misconceptions were identified, particularly among youth players, with a significant proportion providing unsafe responses regarding return-to-play decisions. The study highlights the need for enhanced concussion education, especially for young athletes, and calls for ongoing research and targeted interventions to improve safety in youth ice hockey.

## Introduction

Concussion in sport has become a major concern in the United Kingdom, with an increasing body of evidence demonstrating the adverse long-term health effects of repeated head trauma [1]. For some contact sports, such as rugby, these stories have a direct impact on how the sport is viewed by parents. A report by the Rugby Football Union in 2024 highlighted an existential decline in schools’ rugby participation due in part to parents' concerns over concussion and head injuries [2]. Ice hockey is a fast-paced, contact sport played on a hard playing surface. It involves both intentional (i.e., body checking) and incidental contact that may involve the head. Concussion is a major concern of the ice hockey community in both North America and the United Kingdom [3].

There are 12,236 registered players in the United Kingdom from 53 different ice rinks, including 3,552 youth players. Teams range from youth to university to semi-professional. Youth teams play in 24 leagues, geographically divided into five regions, and range in age from under 10 to under 19 years [4]. The governing bodies in the United Kingdom - England Ice-Hockey and Scottish Ice-Hockey - are well aware of the issues around safety in ice hockey and, in particular, concussion. They have adopted the UK Government Concussion Guidance for Grassroots Sport, which asks players and parents to familiarize themselves with recognizing the signs of concussion and requires clubs to act upon these signs - “if in doubt, sit them out” [5].

There are various definitions for concussion in the research literature, each having its own strengths and weaknesses. We have taken a definition which has been used in previous research on ice hockey concussion with youth players: “A concussion was defined as any mild closed head injury involving altered cognitive functioning (e.g., confusion, memory loss, disorientation), or signs/symptoms (e.g., headache, dizziness, balance problems, nausea), or brief loss of consciousness of no longer than 1 min after a direct or indirect blow to the head” [6].

Though all ages are at risk of concussion, there are particular issues for youth players, i.e., players from ages nine to nineteen years. It is known that the pediatric population is more vulnerable to concussion and experiences more serious short- and long-term symptoms than adults [7,8]. Research in the United States found that 25% of youth players (aged between 16 and 21 years) had sustained at least one concussion over the course of a single season [3]. If anything, this is an underestimate, as it is known that concussions are under-reported [9]. A large body of research has shown that most concussions, at all levels of play, are caused by legal body checking [10,11]. In fact, in both Canada and the United States, there have been calls that body checking should be made illegal in all youth hockey for players under the age of 16 years [12,13]. This is not the case in the United Kingdom, where body checking is legal for children older than 11 years [14]. It is therefore imperative that players and parents understand the signs of concussion and how to manage the risks.

Research in Canada has identified concerns about the knowledge and attitudes of youth ice hockey players toward concussion. Most players were unable to identify all the symptoms of a concussion. In addition, players reported playing while symptomatic and feeling pressure to play while symptomatic. Concussions were often not recognized or not reported as players downplayed their seriousness [15,16]. Other North American research with youth ice hockey players found that there was no significant relationship between concussion knowledge and concussion reporting. Instead, it was the attitudes around taking concussions seriously that were significant predictors of concussion reporting. The research highlights that attitudes toward the significance of concussion must change to achieve meaningful behavioral change [17]. These studies may have limited generalizability to the United Kingdom as there are significant differences in education, healthcare, and sporting culture. 

The only similar research in the United Kingdom was conducted on adult male ice hockey players. This was a small-scale study using the Rosenbaum Concussion Knowledge and Attitude Survey. The research found that the level of the player, i.e., professional or amateur, did not affect their scores on either the Knowledge or Attitude scale. There was, however, a positive correlation between the players' length of experience of playing ice hockey and their knowledge and increasingly safe attitude to concussion. However, the research showed that there remained worryingly unsafe attitudes regarding aspects of the management of concussion [18].

For many sports, such as rugby, schools and teachers play a central role in managing concussions. As ice hockey is played in clubs outside of school, parents, rather than teachers, play a key role in the identification and management of concussion. However, previous research in a number of sports has consistently highlighted issues of concern about parents' understanding of concussion. Parents often do not recognize a concussion, are unaware of the consequences of it, and the protocols in place for its management [19,9]. There have been few studies on the knowledge and attitudes of parents of ice hockey players in North America. For example, a small pilot study in the United States found that parents had a good knowledge of the symptoms of concussion but were less aware of concussion management and policies [20].

The lack of published research on the knowledge of, or attitudes toward, concussion by either youth ice hockey players or their parents in the United Kingdom is a cause of considerable concern and represents a significant gap in our knowledge of how to make the sport safer. We used the validated Rosenbaum Concussion Knowledge and Attitudes Survey - Student Version (RoCKAS-ST) to examine both their knowledge of concussion through the Concussion Knowledge Index (CKI) and their attitudes toward concussion reporting and management through the Concussion Attitudes Index (CAI). We hypothesized that parents would have greater CKI and CAI scores than the youth players. We also hypothesized that there would be a positive correlation between CKI scores and CAI scores; that those with greater knowledge of concussion would have safer attitudes toward concussion. This would be in keeping with the Knowledge-Attitude-Practice model of educational psychology.

## Materials and methods

Study design

The study was a cross-sectional, quantitative survey utilizing the Rosenbaum Concussion Knowledge and Attitudes Survey - Student Version (RoCKAS-ST). The RoCKAS-ST was derived from the original RoCKAS survey, which in turn was based on prior traumatic brain injury surveys [21]. RoCKAS-ST is formulated explicitly for youth populations. This is an internationally validated instrument for concussion research, which has been used across a range of sports, including football, rugby, and ice hockey [22,23]. The RoCKAS-ST has two distinct purposes. The first is examined by the Concussion Knowledge Index (CKI), which assesses both general knowledge of concussion and symptom recognition. The second purpose, the Concussion Attitudes Index (CAI), evaluates attitudes toward concussion reporting and management.

This study did not require formal ethical approval as per the Health Research Authority and Medical Research Council Tool (https://www.hra-decisiontools.org.uk/ethics/). The study was a survey conducted outside the NHS and did not involve any patients. All participants were provided with a study information sheet, which they were required to sign to confirm their consent prior to participation in the survey. In addition, for the youth players, their parents were required to countersign the consent form.

Data collection

The study population was under-16 and under-19 youth ice hockey players and their parents in the United Kingdom. The included players were born from 2008 to 2012. Participants aged 18 years were only included if they were in full-time education. The sampling method used was convenience sampling. The collection period for both lasted six weeks, from October 12, 2025, to November 23, 2025. We utilized two methods of participant recruitment.

Firstly, we contacted the secretaries of youth ice hockey clubs in the United Kingdom and invited them to distribute the survey to the parents of players in the above age groups. This was done using an online platform (surveyplanet.com). We attempted to contact every youth club in the country; however, a few did not have publicly available contact methods. Nevertheless, clubs from all regions of the United Kingdom were contacted with an aim to improve the generalizability of results.

Secondly, we attended local ice hockey matches and directly invited parents and players to participate in the survey. Players and parents were excluded if they had already completed the online survey. Completion of the survey was completely voluntary, and no remuneration was made to any players or parents. The survey took approximately 10 min to complete. Apart from the consent form, no personal identifiers were collected, and participants could withdraw consent at any point prior to the paper being submitted.

The survey includes three internal validity questions to ensure that participants were actively engaged in completing the survey. Participants answering more than one of these questions incorrectly were excluded from the study. Similarly, incompletely answered questionnaires were also excluded from this study.

Data analysis

The RoCKAS-ST contains five parts. Scores from sections 1, 2, and 5 are combined for the Concussion Knowledge Index (CKI). This consists of a combination of true/false questions and a checklist of possible concussion symptoms. Questions scored correctly, or correct symptoms identified, each score one point. The maximum score is 25, and the minimum is zero. Higher scores represent better concussion knowledge.

Sections 3 and 4 combine to form the Concussion Attitudes Index (CAI). This consists of 15 items ranked on a five-point Likert scale. The safest answer scores five points, whereas the least safe answer scores one point. Thus, the range of possible scores is between 15 and 75, with higher scores indicating safer attitudes toward concussion. Questions that score either a four or a five are considered safe answers for the purpose of secondary analysis.

The data from the surveys sent out indirectly via club secretaries and the data obtained directly by attending matches were combined. A total of 33 youth players and 59 parents completed the survey. Four youth players and four parents were excluded on the basis of either incomplete survey results or failing the internal validity questions. Following these exclusions, the survey results from 29 youth players and 55 parents were analyzed.

The data were exported into Microsoft Excel (Microsoft 365; Redmond, WA: Microsoft Corp.) for analysis. The mean scores were calculated separately for the players and parents on the two dimensions - CKI and CAI. These were then compared to assess whether there was a significant difference between players' and parents' scores using an unpaired Student’s t-test. A result was considered significant if p≤0.05. Finally, the Pearson correlation coefficient was used to explore if there was a relationship between the CKI and CAI scores for players and parents.

## Results

Concussion Knowledge Index

The Concussion Knowledge Index yields a score between 0 and 25. The full breakdown of responses is presented in Table 1. The mean CKI score for youth players was 17.86 (SD 3.17), and for parents it was 19.69 (SD 1.77). This demonstrates a statistically significant difference between CKI scores in players and their parents according to a Student's t-test with a value of 3.397 (p=0.0011). The total CKI score was statistically significant despite fewer individual items achieving significance, because the composite score increased statistical power.

**Table 1:** Responses to Sections 1, 2, and 5 which make up the Concussion Knowledge Index (CKI). *P<0.05 is considered statistically significant. P-value is calculated using a Chi-square test.

Questions	Number of players answering correctly	% of players answering correctly	Number of parents answering correctly	% of parents answering correctly	Chi-square value	p-Value
Section 1						
There is a possible risk of death if a second concussion occurs before the first one has healed.	24	83%	49	89%	0.6690	0.4134
People who have had one concussion are more likely to have another concussion.	22	76%	34	62%	1.6853	0.1942
In order to be diagnosed with a concussion, you have to be knocked out.	26	90%	54	98%	3.0440	0.0810
A concussion can only occur if there is a direct hit to the head.	18	62%	46	84%	4.8688	0.0273*
Being knocked unconscious always causes permanent damage to the brain.	23	79%	41	75%	0.2376	0.6259
Symptoms of a concussion can last for several weeks.	23	79%	54	98%	8.8524	0.0029*
Sometimes a second concussion can help a person remember things that were forgotten after the first concussion.	20	69%	46	84%	2.4274	0.1192
After a concussion occurs, brain imaging (e.g., CAT scan, MRI, X-ray, etc.) typically shows visible physical damage (e.g., bruise, blood clot) to the brain.	10	34%	15	27%	0.4722	0.4920
If you receive one concussion and you have never had a concussion before, you will become less intelligent.	27	93%	54	98%	1.4219	0.2331
After 10 days, symptoms of a concussion are usually completely gone.	13	45%	18	33%	1.1940	0.2745
After a concussion, people can forget who they are and not recognize others, but be perfect in every other way.	15	52%	36	65%	1.5008	0.2205
Concussions can sometimes lead to emotional disruptions.	21	72%	51	93%	6.3987	0.0114*
An athlete who gets knocked out after getting a concussion is experiencing a coma.	11	38%	5	9%	10.2425	0.0014*
There is rarely a risk to long-term health and well-being from multiple concussions.	19	66%	48	87%	5.5674	0.0183*
Section 2						
While playing in a game, Player Q and Player X collide with each other and each suffers a concussion. Player Q has never had a concussion in the past. Player X has had 4 concussions in the past. It is likely that Player Q’s concussion will affect his long-term health and well-being.	22	76%	33	60%	2.1135	0.1460
While playing in a game, Player Q and Player X collide with each other and each suffers a concussion. Player Q has never had a concussion in the past. Player X has had 4 concussions in the past. It is likely that Player X’s concussion will affect his long-term health and well-being.	22	76%	46	84%	0.7443	0.3883
Player F suffered a concussion in a game. She continued to play in the same game despite the fact that she continued to feel the effects of the concussion. Even though Player F is still experiencing the effects of the concussion, her performance will be the same as it would be had she not suffered a concussion.	19	66%	49	89%	6.8433	0.0089*
Section 5						
Headache	28	97%	55	100%	1.9194	0.1659
Sensitivity to light	22	76%	53	96%	8.3428	0.0039*
Difficulty remembering	21	72%	52	95%	8.1725	0.0043*
Drowsiness	19	66%	50	91%	8.3462	0.0039*
Feeling in a “fog”	22	76%	49	89%	2.5403	0.1110
Feeling slowed down	16	55%	43	78%	4.8090	0.0283*
Difficulty concentrating	28	97%	47	85%	2.4443	0.1180
Dizziness	27	93%	55	100%	3.8856	0.0487*

Concussion Attitudes Index

The Concussion Attitudes Index (CAI) gives a score between 15 and 75. The full breakdown of responses can be found in Table 2. The mean CAI score for youth players was 57.41 (76.5% of the maximum possible score of 75, SD: 8.15). This was compared to a mean score of 62.62 (83.5%, SD: 6.17) for the parents. This difference was again judged to be statistically significant according to a Student's t-test with a score of 3.280 (p=0.0015).

**Table 2:** Responses to Sections 3 and 4 which make up the Concussion Attitudes Index (CAI).

Questions	Number of players giving safe answer	% of players giving safe answers	Number of parents giving safe answers	% of parents giving safe answers
Section 3				
I would continue playing a sport while also having a headache that resulted from a minor concussion.	12	41%	40	73%
I feel that coaches need to be extremely cautious when determining whether an athlete should return to play.	21	72%	52	95%
I feel that concussions are less important than other injuries.	22	76%	49	89%
I feel that an athlete has a responsibility to return to a game even if it means playing while still experiencing symptoms of a concussion.	22	76%	53	96%
I feel that an athlete who is knocked unconscious should be taken to the emergency room.	26	90%	54	98%
Section 4				
Player R suffers a concussion during a game. Coach A decides to keep Player R out of the game. Player R’s team loses the game. I feel that coach A made the right decision to keep player R out of the game.	25	86%	52	95%
Player R suffers a concussion during a game. Coach A decides to keep player R out of the game. Player R’s team loses the game. Most athletes would feel that coach A made the right decision to keep player R out of the game.	22	76%	41	75%
Athlete M suffered a concussion during the first game of the season. Athlete O suffered a concussion of the same severity during the semifinal playoff game. Both athletes had persisting symptoms. I feel that athlete M should have returned to play during the first game of the season.	21	72%	50	91%
Athlete M suffered a concussion during the first game of the season. Athlete O suffered a concussion of the same severity during the semifinal playoff game. Both athletes had persisting symptoms. Most athletes would feel that athlete M should have returned to play during the first game of the season.	17	59%	34	62%
Athlete M suffered a concussion during the first game of the season. Athlete O suffered a concussion of the same severity during the semifinal playoff game. Both athletes had persisting symptoms. I feel that athlete O should have returned to play during the semifinal playoff game.	18	62%	42	76%
Athlete M suffered a concussion during the first game of the season. Athlete O suffered a concussion of the same severity during the semifinal playoff game. Both athletes had persisting symptoms. Most athletes feel that athlete O should have returned to play during the semifinal playoff game.	13	45%	31	56%
Athlete R suffered a concussion. Athlete R’s team has an athletic trainer on the staff. I feel that the athletic trainer, rather than athlete R, should make the decision about returning athlete R to play.	18	62%	34	62%
Athlete R suffered a concussion. Athlete R’s team has an athletic trainer on the staff. Most athletes would feel that the athletic trainer, rather than athlete R, should make the decision about returning athlete R to play.	15	52%	26	47%
Athlete H suffered a concussion and he has a game in two hours. He is still experiencing symptoms of concussion. However, athlete H knows that if he tells his coach about the symptoms, his coach will keep him out of the game. I feel that athlete H should tell his coach about the symptoms.	24	83%	49	89%
Athlete H suffered a concussion and he has a game in two hours. He is still experiencing symptoms of concussion. However, athlete H knows that if he tells his coach about the symptoms, his coach will keep him out of the game. Most athletes would feel that athlete H should tell his coach about the symptoms.	23	79%	38	69%

Relationship between CKI and CAI scores

**Figure 1:**
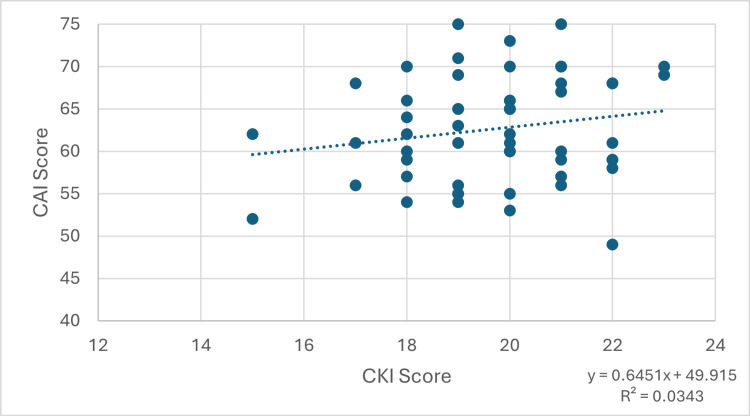
Graph showing the relationship between CKI score and CAI score in the parents group. CKI: Concussion Knowledge Index; CAI: Concussion Attitudes Index

For parents, the Pearson correlation coefficient was 0.1852, indicating a weak positive correlation between knowledge and attitude scores (95% confidence interval: -0.0842 to 0.4294). A corresponding t-test produced a p-value of 0.1758, indicating no statistical significance. Figure 1 demonstrates this relationship.

**Figure 2:**
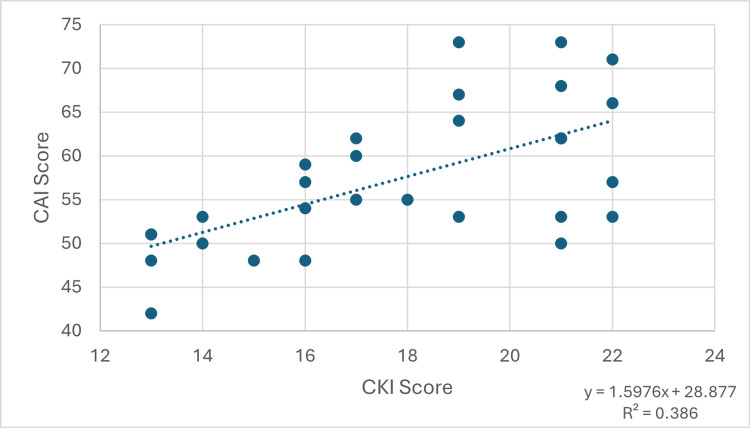
Graph showing the relationship between CKI score and CAI score in the youth players. CKI: Concussion Knowledge Index; CAI: Concussion Attitudes Index

For youth players, the Pearson correlation coefficient was 0.6213, indicating a strong positive correlation between knowledge and attitude scores (95% confidence interval: 0.3299 to 0.8046). This was statistically significant (p=0.00032). This relationship is demonstrated in Figure 2.

## Discussion

Both players and their parents scored relatively well on the Concussion Knowledge Index. Among the players, the mean score was comparable to that of Canadian elite youth ice hockey players (72% correct in North America; 71% correct in our study) and higher than youth players in other contact sports [24,25]. Parents demonstrated similarly high scores when compared with findings from comparable North American studies [8].

However, the range of scores within the youth section of the study was large (12-22), suggesting a highly variable level of knowledge about concussion. Within our small sample size, a third of players felt that there is rarely a risk to long-term health and well-being from multiple concussions. This raises concerns about awareness of the long-term detrimental impact of multiple concussions. This finding may, in part, explain why attitudes toward return to play after a concussion were often more haphazard - players know about concussions, but because of a lack of insight into the potential adverse effects, they are more likely to ignore the guidelines and return to play sooner than they should. This predisposes athletes to risks of further concussions and, therefore, may have important implications regarding secondary prevention.

Another question that was consistently poorly answered was regarding imaging findings in concussed players. Only 34% of players and 27% of parents answered this question correctly. One of the hallmarks of concussion is that, in most cases, there are no abnormalities found on advanced imaging such as computed tomography or magnetic resonance imaging, yet most respondents were unaware of this [21]. This has important implications for clinicians working in the emergency department, who are often the first point of contact for players presenting after a head injury. A normal neuroimaging result does not exclude the diagnosis of concussion, and emergency clinicians therefore have a crucial role in educating patients and their families about this distinction. Comprehensive safety-netting advice should be routinely provided irrespective of imaging results, including information on expected symptoms, warning signs that warrant urgent reassessment, guidance on cognitive and physical rest, and return-to-play guidance.

Parents performed exceptionally well on Section 5, which assessed concussion symptom recognition. Participants were asked to identify concussion symptoms from a list of 16 options, eight of which were correct. In this section, parents outperformed youth players across seven of the eight variables. Recognition of common symptoms was high, with 100% of parents and over 90% of players correctly identifying headache and dizziness as signs of concussion. However, a notable proportion of incorrect responses remained, most commonly reduced breathing rate and panic attacks. Overall, these findings are encouraging and demonstrate good recognition of concussion symptoms. This is important in the context of UK concussion policy, including the UK-wide Concussion Guidelines for Grassroots Sport, which emphasize early recognition, immediate removal from play, and appropriate follow-up. As parents are often the first adults to observe children after sporting activity, high parental awareness increases the likelihood of prompt identification and reduces the risk of continued participation while symptomatic, thereby helping to prevent further injury.

Overall, in the concussion knowledge section, the mean score for parents (19.69) was higher than that for youth players (17.86), a difference that was statistically significant. This is likely due to a number of factors, which may include increased general health literacy, increased lived experience, including personal experience of concussions, and the fact that concussion guidelines are often tailored to adult audiences. This highlights the need for improved concussion education among the ice hockey community in the United Kingdom, especially among youth players, and suggests that current measures by England Ice-Hockey and Scottish Ice-Hockey are insufficient. In particular, formal concussion sessions with educational material targeted at youth audiences could be considered.

In comparison to the well-scored Concussion Knowledge Index, the Concussion Attitudes Index results were more variable. The mean score among youth players was 57.41. This score is lower than that of school rugby players in Ireland but similar to that of youth ice hockey players in Canada [26,24]. The mean score among parents was 62.62, comparable to scores in other adult populations studied in the United Kingdom [27,28].

Questions that scored either four or five were considered safe answers. Youth players gave a mean of 10.31 safe answers (out of 15), which means almost one-third of the answers on the Concussion Attitudes Index completed by youth hockey players were unsafe. Of note, only 41% of players said that they would not play sport while having a headache that resulted from a minor concussion, and 38% felt that an athlete with persisting symptoms from a concussion should return to play if it is a semifinal. This is in clear violation of all recognized concussion protocols, including those which are recommended by the English Ice-Hockey Association, and suggests a distinct barrier between what players know and how they feel or act. The underlying reasons for this are understudied in ice hockey but may include competitive pressure, fear of being removed from play, a desire not to let down teammates, peer pressure, and social norms of playing through injury. Further qualitative data is needed to evaluate this.

As expected, parents provided a higher proportion of safe answers (mean 11.73 out of 15) compared to players. However, 38% of parents believed the decision to return to sport should rest with the player rather than a qualified professional, such as an athletic trainer or physiotherapist. This may reflect trust in their child’s judgment or a desire to respect their autonomy, particularly for older youth athletes. Alternatively, it could reflect a limitation of the survey - in the United Kingdom, youth ice hockey, qualified professionals such as athletic trainers or physiotherapists are rarely present at matches, with first aid responsibility often falling to rink staff or other parents who may lack formal concussion training. In this context, parents may feel their child is better positioned to assess readiness to return. Of additional concern, 10% of parents still viewed concussion as less critical than other injuries - a worrying finding given the wide-ranging physical, psychological, and cognitive complications that can arise from under-recognized or undertreated concussions.

Overall, in the concussion attitudes portion of the survey, the mean score of the parents (62.62) was higher than that of the youth players (57.41), and this was statistically significant. Practically, this finding suggests that targeted educational interventions for young athletes are needed to ensure that their attitudes support safe reporting, adherence to medical advice, and reduced risk of repeat or prolonged injury.

Notably, the correlation between knowledge and attitudes was significant among players but not among their parents. This could be due to numerous factors that need further research. Parents may feel pressured to express “correct” attitudes about concussion safety regardless of their actual knowledge. This artificially elevates attitude scores and weakens the knowledge-attitude correlation. Players - especially youth players in this age bracket - may respond more authentically, strengthening the link. Young players may be naturally more inclined to take risks, and it is only with increasing knowledge of the harmful effects of concussion that safer attitudes prevail.

Limitations

There are several limitations to this study. Firstly, as a cross-sectional survey, there is an inherent risk of selection bias. Although part of the survey was administered anonymously online, which may have reduced this risk to some extent, it is likely that players or parents who were already interested in or knowledgeable about concussions were more inclined to participate. Convenience sampling at matches may have led to over-representation of certain groups, such as clubs located in the authors’ region. These factors could lead to biased estimates and limit the generalizability of the findings to the wider ice hockey population. Secondly, the study focused primarily on players aged 14-18 years. Previous research has demonstrated that concussion prevalence in younger age groups can be as high, if not higher, than in older adolescents, and therefore, the extent to which these results apply to younger players remains uncertain. Thirdly, the survey was anonymized to limit response bias; however, the Concussion Attitudes Index reflects what players believe they should do, which does not necessarily correspond to what they would do in real-world scenarios. Further research incorporating prospective studies is needed to address this gap.

Furthermore, the sample size was relatively small (55 parents and 29 players); therefore, further research could aim to obtain a much larger sample to aid in increasing confidence in the results and their generalizability. Finally, the RoCKAS-ST survey was developed in North America, and several items are culturally specific. Questions about cleats or athletic trainers may have been confusing for a British audience, potentially limiting participants’ ability to respond accurately and affecting the overall validity of the results.

## Conclusions

This study evaluated concussion knowledge and attitudes among youth ice hockey players and their parents in the United Kingdom. Overall, both groups demonstrated good levels of concussion knowledge, with scores comparable to those reported in the literature. However, substantial variability existed, and important misconceptions were identified in both groups. The limited number of “safe” answers among the player cohort on the Concussion Attitudes Index was of particular concern.

Concussions remain a significant health concern in youth ice hockey, where high-speed play and player contact increase the risk of head injury. Evidence consistently shows that young athletes are more susceptible to prolonged recovery and potential long-term effects, underscoring the importance of early recognition, proper management, and conservative return-to-play protocols. Continued education for players, parents, and coaches, combined with ongoing research, will help create a safer environment that allows young athletes to enjoy the sport while minimizing preventable brain injuries.
